# Genome-wide identification and comprehensive characterization of the ADF gene family in *Triticum monococcum* L. subsp. *aegilopoides* with insights into structure, evolution and cold stress response

**DOI:** 10.3389/fpls.2025.1649202

**Published:** 2025-08-04

**Authors:** Xin Liu, Minghu Zhang, Jian Su, Lei Wu, Mang Shen, Qi Wang, Yamei Zhuang, Lianquan Zhang, Haosheng Li, Gang Chen

**Affiliations:** ^1^ Faculty of Agriculture, Forestry and Food Engineering, Yibin University, Yibin, Sichuan, China; ^2^ Crop Research Institute, Shandong Academy of Agricultural Sciences, Jinan, Shandong, China; ^3^ State Key Laboratory of Crop Gene Exploration and Utilization in Southwest China, Sichuan Agricultural University, Chengdu, Sichuan, China; ^4^ Key Laboratory of Aquatic Genomics, Ministry of Agriculture and Rural Affairs, Beijing Key Laboratory of Fishery Biotechnology, Chinese Academy of Fishery Sciences, Beijing, China

**Keywords:** *Triticum monococcum* L. subsp. *aegilopoides*, ADF, evolution, expression profiles, cold stress

## Abstract

Actin-depolymerizing factors (ADFs) play crucial roles in cytoskeletal dynamics and stress adaptation in plants. In this study, we identified nine ADF genes (*TbADF1* to *TbADF9*) in *Triticum monococcum* L. subsp. *aegilopoides*. Chromosomal distribution analysis revealed that these genes are unevenly distributed across five chromosomes, with evidence of tandem duplication events. Phylogenetic analysis clustered the *TbADFs* into four subfamilies, indicating evolutionary conservation among wheat relatives. Gene structure and motif analyses confirmed the presence of a conserved ADF domain. Additionally, promoter region analysis revealed a variety of cis-regulatory elements associated with hormone signaling and stress responses. Predictions of binding pockets and protein–protein interaction networks indicated potential functional sites and interactions with cytoskeletal regulators. Codon usage bias analysis showed a preference for GC-rich codons, which may enhance translation efficiency under stress. Codon usage bias analysis indicated GC-rich optimization, potentially enhancing translation efficiency under stress. Promoter methylation levels ranged from 0.0907 to 0.3053, suggesting that epigenetic regulation may contribute to the control of gene expression. Transcriptomic profiling across six tissues and under cold stress conditions (4°C for 24 hours) revealed both tissue-specific expression patterns and differential cold responses. Notably, *TbADF1*, *TbADF4*, *TbADF6*, and *TbADF7* were upregulated, with *TbADF6* exhibiting the strongest induction, as its TPM value increased from 29.07 to 300.01. Furthermore, co-expression and gene ontology enrichment analyses of the upregulated genes identified key biological pathways involved in membrane integrity, phosphorylation, ribosome maturation, and lipid signaling. These findings highlight the central role of *TbADF6* in cold adaptation.

## Introduction

*Triticum monococcum* L. subsp. *aegilopoides* is recognized as one of the earliest domesticated cereal crops, with its cultivation dating back approximately 10,000 years to the Fertile Crescent (Ahmed et al., 2023). As an ancient wheat species, it exhibits remarkable resilience compared to modern common wheat, particularly in its tolerance to abiotic stresses such as drought, low temperatures, and nutrient-deficient soil (Vanková et al., 2014). This resilience is largely attributed to its rich genetic diversity, making wild einkorn wheat a valuable resource for introgressing stress-tolerance traits into modern wheat cultivars (Liu et al., 2015; Tounsi et al., 2016; Zhang et al., 2021a). Although its cultivation has substantially declined in modern agriculture, wild einkorn wheat remains a critical component of global wheat germplasm collections. It provides essential genetic material for breeding programs aimed at improving yield stability and environmental adaptability, particularly in the face of ongoing climate change (Mondal et al., 2016).

Actin-depolymerizing factor (ADF) is a highly conserved, small actin-binding protein ubiquitously present in eukaryotic organisms. Initially identified in chicken brain tissue (Bamburg et al., 1980), ADF has since been found to be widely distributed across plant species (Inada, 2017). ADF proteins are central to regulating actin cytoskeleton dynamics by binding to actin monomers (G-actin) and filaments (F-actin), thereby promoting severing, depolymerization, and bundling (Bernstein and Bamburg, 2010; Du et al., 2016). These activities are essential for maintaining cellular structure and support diverse physiological processes, including cell polarity, proliferation, division, intracellular trafficking, signal transduction, and pathogen recognition (Drøbak et al., 2004). Furthermore, ADFs play important roles in plant responses to abiotic stresses such as cold and drought, underscoring their significance in enhancing plant adaptability and resilience under adverse environmental conditions.

Low-temperature stress is a major constraint on crop productivity, as it inhibits plant growth, delays development, and, in severe cases, causes tissue damage or regression, ultimately leading to yield losses (Song et al., 2017; Hassan et al., 2021). Increasing evidence indicates that ADF genes play a critical role in mediating plant responses to cold stress through dynamic regulation of the cytoskeleton. In maize, for instance, 15 ADF genes exhibit tissue-specific and stimulus-responsive expression patterns, suggesting functional diversification (Yang et al., 2024). In *A. thaliana*, knockout mutants lacking *AtADF5* show significantly reduced survival under low-temperature conditions, highlighting the essential role of ADFs in cold tolerance (Zhang et al., 2021b). Similar patterns have been observed in wheat, where cold-tolerant cultivars display specific upregulation of ADF genes in response to low temperatures, whereas cold-sensitive varieties show minimal expression changes (Ouellet et al., 2001). A genome-wide analysis of wheat identified 25 ADF genes, seven of which are significantly regulated by cold stress. Notably, heterologous expression of the wheat gene *TaADF16* in *Arabidopsis* enhanced cold tolerance, suggesting its potential utility in transgenic approaches to improve stress resilience (Xu et al., 2021).

Although modern wheat breeding has substantially increased yield and grain quality, it has also led to a significant reduction in the genetic diversity of cultivated varieties, often compromising stress-resistance alleles. To address this limitation, growing attention has been directed toward wheat wild relatives and landraces, such as wild einkorn wheat, which serve as reservoirs of untapped genetic variation (Riar et al., 2021). In this study, we utilized the reference genome of wild einkorn wheat to identify nine ADF genes. These genes were systematically characterized with respect to their gene structures, conserved motifs, and expression patterns across different tissues and under cold stress. Our findings not only enhance the understanding of ADF gene family evolution and function in early domesticated wheat but also offer valuable genomic resources for breeding programs targeting improved cold tolerance. Ultimately, these insights contribute to the broader goal of promoting sustainable wheat production under increasingly variable environmental conditions.

## Materials and methods

### Plant materials

*Triticum monococcum* L. subsp. *aegilopoides*, accession G52, an ancient and resilient crop with considerable potential for improving stress tolerance in modern wheat, was employed to investigate the role of ADF genes in response to cold stress. Seeds were kindly provided by Professor Lianquan Zhang (Sichuan Agricultural University). To break dormancy, seeds were stratified at 4°C for 24 hours, followed by germination at room temperature for 7 days. Subsequently, ten uniform seedlings were subjected to cold treatment at 4°C for 24 hours, while another set of ten seedlings was maintained under control conditions. Leaf tissues from both treated and control groups were collected, with three biological replicates per group. Samples were immediately flash-frozen in liquid nitrogen and stored at −80°C for subsequent analyses.

### Identification of ADF genes in *Triticum monococcum* L. subsp. *aegilopoides*

The reference genome of *Triticum monococcum* L. subsp. *aegilopoides* TA299 was obtained from the DRYAD repository (https://doi.org/10.5061/dryad.v41ns1rxj). The ADF domain (Pfam accession: PF00241) was retrieved from the Pfam database (http://pfam.xfam.org/). A Hidden Markov Model (HMM) search was performed using HMMER v3.0 (Mistry et al., 2013) with a stringent E-value threshold of ≤ 1e−10 to identify candidate ADF protein sequences. The presence of the ADF domain in these sequences was further validated using the NCBI Conserved Domain Database (CDD; https://www.ncbi.nlm.nih.gov/cdd/), SMART (http://smart.embl-heidelberg.de/), and Pfam. Physicochemical properties of the identified *TbADFs*, including molecular weight, isoelectric point, and instability index, were calculated using the ExPASy ProtParam tool (https://web.expasy.org/protparam/). Subcellular localization of *TbADFs* were predicted using WoLF PSORT (https://wolfpsort.hgc.jp/) and Cell-PLoc 2.0(http://www.csbio.sjtu.edu.cn/bioinf/Cell-PLoc-2/).

### Chromosomal localization, phylogenetic analysis, and collinearity analysis of the *TbADFs*

Positional information for ADF genes was extracted from the GFF3 annotation file of the *Triticum monococcum* L. subsp. aegilopoides reference genome. Chromosomal locations of the identified ADF genes were visualized using TBtools v2.097 (Chen et al., 2023). A phylogenetic tree was constructed based on full-length ADF protein sequences from eight plant species—*Arabidopsis thaliana*, *Aegilops tauschii*, *Oryza sativa*, *Zea mays*, *Triticum urartu*, *Triticum turgidum*, *Triticum aestivum*, and *Triticum monococcum* L. subsp. *aegilopoides*—using the Maximum Likelihood (ML) method in IQ-TREE v2.0 (Nguyen et al., 2015). The best-fit amino acid substitution model was determined automatically using ModelFinder within IQ-TREE, and tree robustness was assessed with 1,000 bootstrap replicates. To investigate evolutionary relationships and genomic conservation, collinearity analysis between *Triticum monococcum* L. subsp. *aegilopoides* and three related wheat species (*T. urartu*, *T. turgidum*, and *T. aestivum*) was performed using MCScanX (Wang et al., 2012) with default parameters.

### Analysis of conserved motifs and cis-acting elements in the *TbADFs*

Gene structure information for the ADF genes was extracted from the GFF3 annotation file of the *Triticum monococcum* L. subsp. *aegilopoides* reference genome. Conserved motifs within the *TbADFs* protein sequences were identified using the MEME Suite (http://meme-suite.org/meme), with the maximum number of motifs set to 10 and other parameters kept at default settings. To investigate potential regulatory mechanisms, the 2,000 bp upstream regions from the transcription start sites of the *TbADFs* were extracted as putative promoter sequences. These promoter regions were analyzed for cis-acting regulatory elements using the PlantCARE database (http://bioinformatics.psb.ugent.be/webtools/plantcare/html/) (Lescot et al., 2002).

### Protein 3D structure modeling and binding pocket prediction

The tertiary structure of the *TbADFs* were predicted using AlphaFold2. The predicted 3D structure was subsequently visualized and analyzed using PyMOL v2.5.5 (DeLano, 2002), facilitating detailed examination of the protein’s spatial conformation. To further investigate the surface topology and potential functional sites, the CASTp 3.0 web server (http://sts.bioe.uic.edu/castp/index.html?2pk9) (Tian et al., 2018) was utilized to identify and characterize surface pockets and cavities at multiple scales, offering insights into possible ligand-binding regions and structural features relevant to *TbADFs*.

Codon usage bias was assessed using CodonW (version 1.4.2; http://codonw.sourceforge.net/), with several key indices calculated to characterize codon usage patterns. These included the effective number of codons (ENC), codon adaptation index (CAI), relative synonymous codon usage (RSCU), overall genomic GC content, GC content at the third codon position (GC3s), and nucleotide frequencies at the third synonymous codon position (T3s, C3s, A3s, G3s). To further investigate the factors shaping codon usage bias, Parity Rule 2 (PR2) analysis was performed to explore the relative contributions of mutational pressure and natural selection. Additionally, ENC-GC3s plots were generated to evaluate the extent of codon bias across genes. All visualizations, including the ENC and PR2 plots, were created using the Matplotlib library (Barrett et al., 2005) to ensure clear graphical representation of the results.

### Protein interaction network prediction

The *TbADFs* amino acid sequences from *Triticum monococcum* L. subsp. *aegilopoides* were submitted to the STRING database (http://www.string-db.org/) (Szklarczyk et al., 2021) to construct a protein-protein interaction (PPI) network, with *A. thaliana* selected as the reference organism due to its well-characterized protein interaction data. The analysis was performed using default parameters, including a medium confidence score threshold (0.6) to predict functional associations based on evidence such as co-expression, experimental data, and conserved co-occurrence. The resulting PPI network was visualized to identify potential interacting partners of *TbADFs* and to infer their functional roles in cellular processes.

### DNA methylation frequency calculation

To evaluate DNA methylation levels in *TbADFs*, we quantified methylation frequencies in both promoter and gene body regions using publicly available whole-genome bisulfite sequencing data for *Triticum monococcum* L. subsp. *aegilopoides* accession TA299, obtained from the Dryad Digital Repository (https://datadryad.org/dataset/doi:10.5061/dryad.v41ns1rxj). Promoter regions were defined as the 2 kilobases upstream of the transcription start site (TSS), while gene body regions included the entire transcribed sequence excluding the promoter.

For each *TbADFs* and genomic region, methylation frequency was calculated as the ratio of methylated CpG sites to the total number of CpG sites (methylated + unmethylated), using the following formula:


$$\text{Methylation Frequency}=\frac{\text{Number of methylated CpG sites}}{\text{Total number of CpG sites}}$$

Methylation calls were extracted from the downloaded data files, and region-specific methylation frequencies were computed using custom Python scripts. These scripts parsed CpG methylation count data and calculated the average methylation level across all CpG sites within each defined region for each gene.

### Tissue-specific and cold stress-induced expression analysis of *TbADFs*

Transcriptome data for *Triticum monococcum* L. subsp. *aegilopoides* from various tissues and cold stress treatments were retrieved from the NCBI Sequence Read Archive (SRA) under accession numbers PRJEB61155 and PRJEB21284. Adapter sequences and low-quality reads were removed using Trimmomatic (v0.39) (Bolger et al., 2014). The resulting high-quality reads were pseudo-aligned to the reference genome and quantified using Kallisto (v0.46.2) (Bray et al., 2016) to estimate gene expression levels. A heatmap was generated using TBtools (v2.097) (Chen et al., 2023) to visualize expression patterns. To explore gene co-expression relationships, Pearson correlation analysis was performed between four upregulated *TbADFs* and all other expressed genes. Raw p-values from these pairwise correlation tests were adjusted for multiple comparisons using the Benjamini-Hochberg procedure to control the false discovery rate (FDR) at 5%. Genes with a correlation coefficient greater than 0.9 and a p-value less than 0.001 were first identified as candidate highly co-expressed, these were subsequently subjected to Benjamini-Hochberg correction, and pairs with an FDR-adjusted p-value less than 0.05 were considered highly co-expressed. Co-expression networks and conserved motif distributions were visualized using Cytoscape (v3.10.2) (Shannon et al., 2003). Gene Ontology (GO) enrichment analysis was conducted to infer the potential biological functions of the co-expressed genes, with GO annotations obtained from the KOBAS database (http://bioinfo.org/kobas) (Xie et al., 2011). The enrichment results were visualized using appropriate R packages.

### Real-time RT-PCR

Total RNA was extracted from *Triticum monococcum* L. subsp. *aegilopoides* using the RNAprep Pure Plant Kit (Tiangen, China), following the manufacturer’s instructions. First-strand cDNA was synthesized from 1 µg of total RNA using the RevertAid First Strand cDNA Synthesis Kit (Thermo Scientific, USA). Gene-specific primers for the *TbADF* gene family (**Supplementary Table S1**) were designed using Primer 5 software. Quantitative real-time PCR (qRT-PCR) was conducted on a Veriti 96-Well Thermal Cycler (Applied Biosystems, USA), with *actin* used as the internal reference gene for normalization. Each 10 µl reaction contained 5 µl of 2× TB Green Premix Ex Taq II (Tli RNaseH Plus; Takara, Japan), 0.4 µl of each primer (10 µM), 1 µl of cDNA, and 3.2 µl of sterile double-distilled water. The amplification protocol consisted of an initial denaturation at 95°C for 3 min, followed by 39 cycles of 95°C for 10 s and 58°C for 30 s. A melt curve analysis was performed to verify amplification specificity, using a ramp from 65°C to 95°C in 0.5°C increments after denaturation at 95°C for 5 s. Relative expression levels of *TbADFs* were calculated using the 2^−ΔΔCt^ method.

### Subcellular localization

Clone the CDS sequence of *TbADF6* from *Triticum monococcum* L. subsp. *aegilopoides* cDNA. Digest the pCAMBIA2300-GFP vector with BamHI, and insert the amplified product into the linearized pCAMBIA2300-GFP vector using the pEASY^®^-Basic Seamless Cloning and Assembly Kit. Verify the recombinant construct by DNA sequencing. Transform the construct into *Agrobacterium*. Resuspend and mix the Agrobacterium cultures, then infiltrate them into tobacco leaves. After 36 hours of incubation, observe the fluorescence using a confocal laser scanning microscope.

## Results

### Identification of ADFs in *Triticum monococcum* L. subsp. *aegilopoides*

To identify ADF genes in *Triticum monococcum* L. subsp. *aegilopoides*, BLASTP searches were performed using known ADF protein sequences from *A. thaliana*, *O. sativa*, and *Zea mays* as queries against the *Triticum monococcum* L. subsp. *aegilopoides*. Redundant sequences were filtered using HMM profiling, resulting in the identification of nine unique ADF proteins, designated *TbADF1* through *TbADF9* (**Supplementary Table S2**). These proteins ranged in length from 138 to 270 amino acids, with molecular weights between 16.08 and 30.19 kDa and isoelectric points (pI) from 4.81 to 9.44. Six of the proteins were acidic (pI< 7). The instability index ranged from 33.17 to 56.08, indicating that six proteins may be unstable (instability index > 40). The aliphatic index varied from 58.17 to 80.52, and all nine proteins had negative GRAVY (grand average of hydropathicity) scores, suggesting a hydrophilic nature. Subcellular localization predictions indicated that all *TbADFs* are cytoplasmic.

### Chromosomal distribution and evolutionary analysis of the *TbADFs*

**Figure 1:**
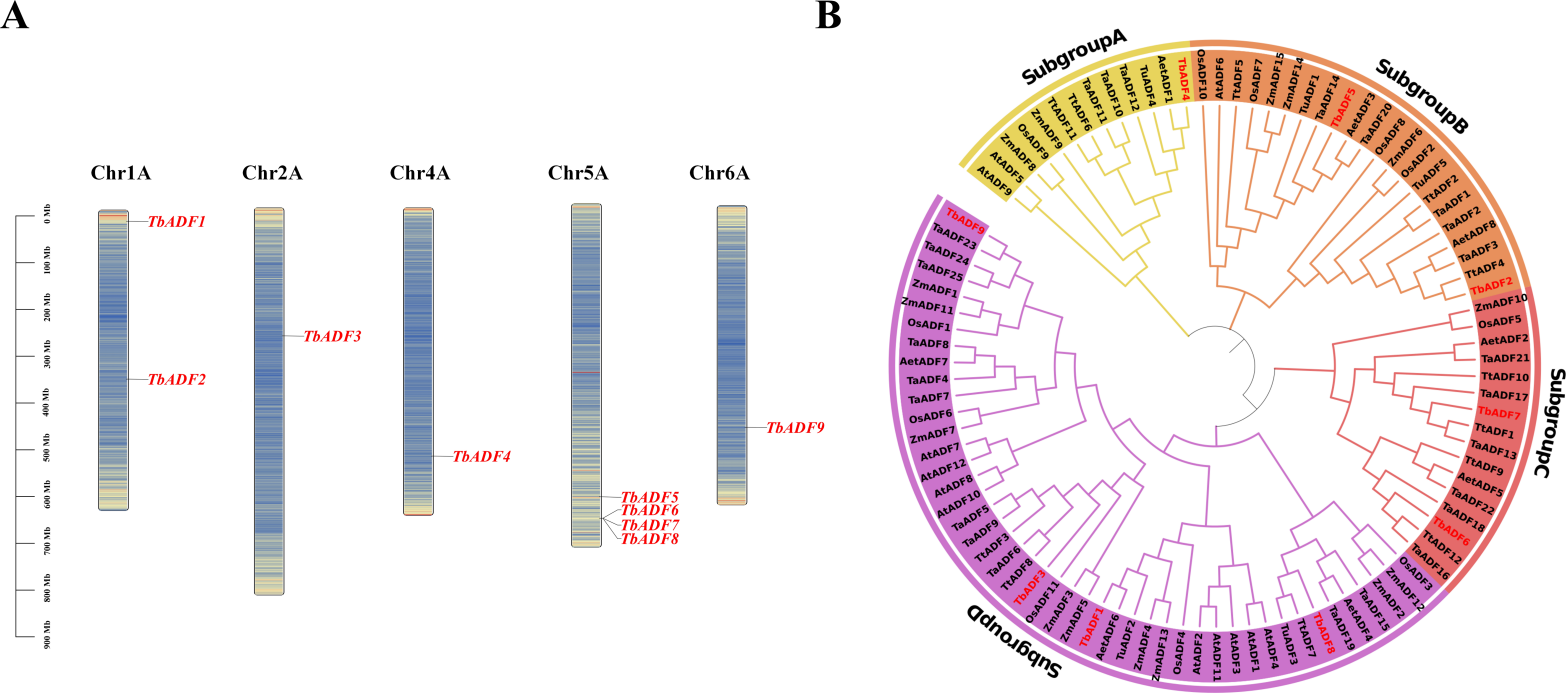
Localization and phylogenetic analysis of ADF genes. **(A)** Distribution of ADF genes across chromosomes, with positions indicated by colored bars. **(B)** Phylogenetic tree of ADF family proteins from multiple species.

Chromosomal mapping revealed that the nine *TbADFs* are unevenly distributed across five chromosomes. Specifically, chromosomes 2A, 4A, and 6A each contain one gene, while chromosome 1A has two genes, and chromosome 5A carries four (**Figure 1A**). Notably, *TbADF6*, *TbADF7*, and *TbADF8* are clustered in adjacent genomic regions, suggesting possible tandem duplication events or localized selective pressures. To assess the evolutionary relationships among *TbADFs*, a phylogenetic tree was constructed using 97 ADF protein sequences from eight plant species. These ADFs clustered into four subfamilies (subgroups A–D), each containing 13 to 46 members. The *TbADFs* are represented in all four subgroups: one gene in subgroup A, two genes each in subgroups B and C, and four genes in subgroup D (**Figure 1B**).

**Figure 2:**
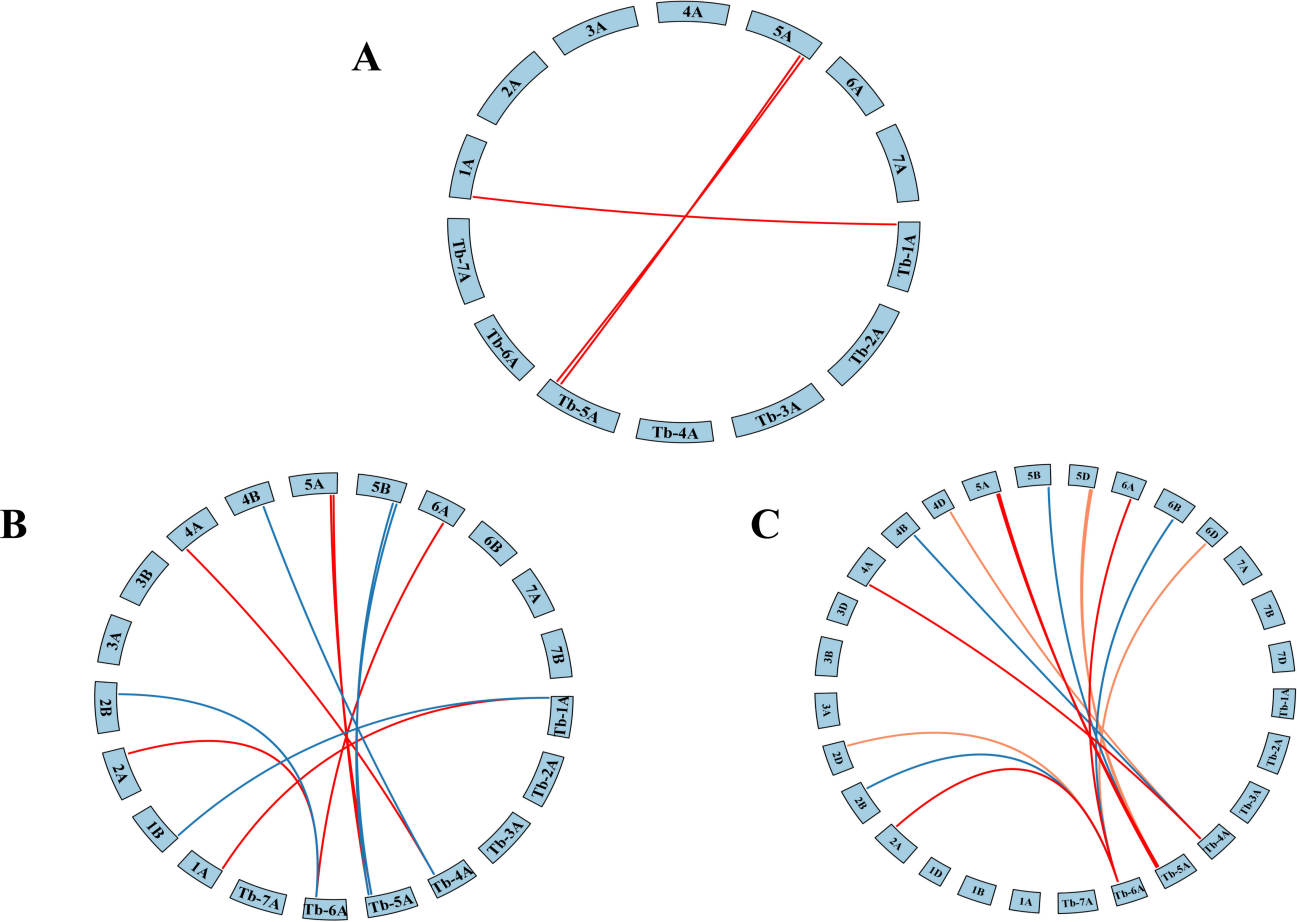
Collinearity analysis of *Triticum monococcum* L. subsp. *aegilopoides* with different ploidy levels of wheat: **(A)** *Triticum monococcum* L. subsp. *aegilopoides-Triticum urartu*, **(B)** *Triticum monococcum* L. subsp. *aegilopoides-Triticum turgidum*, **(C)** *Triticum monococcum* L. subsp. *aegilopoides -Triticum aestivum*.

To further explore ADF gene conservation, synteny analysis was performed between *Triticum monococcum* L. subsp. *aegilopoides* and three wheat relatives of varying ploidy levels: diploid *Triticum urartu*, tetraploid *Triticum turgidum*, and hexaploid *Triticum aestivum*. This analysis identified 3, 12, and 16 orthologous gene pairs, respectively (**Figures 2A–C**), suggesting that ADF genes have been conserved through wheat evolution and polyploidization, likely under purifying selection.

### Analysis of conserved motifs and cis-acting regulatory elements of *TbADFs*

**Figure 3:**
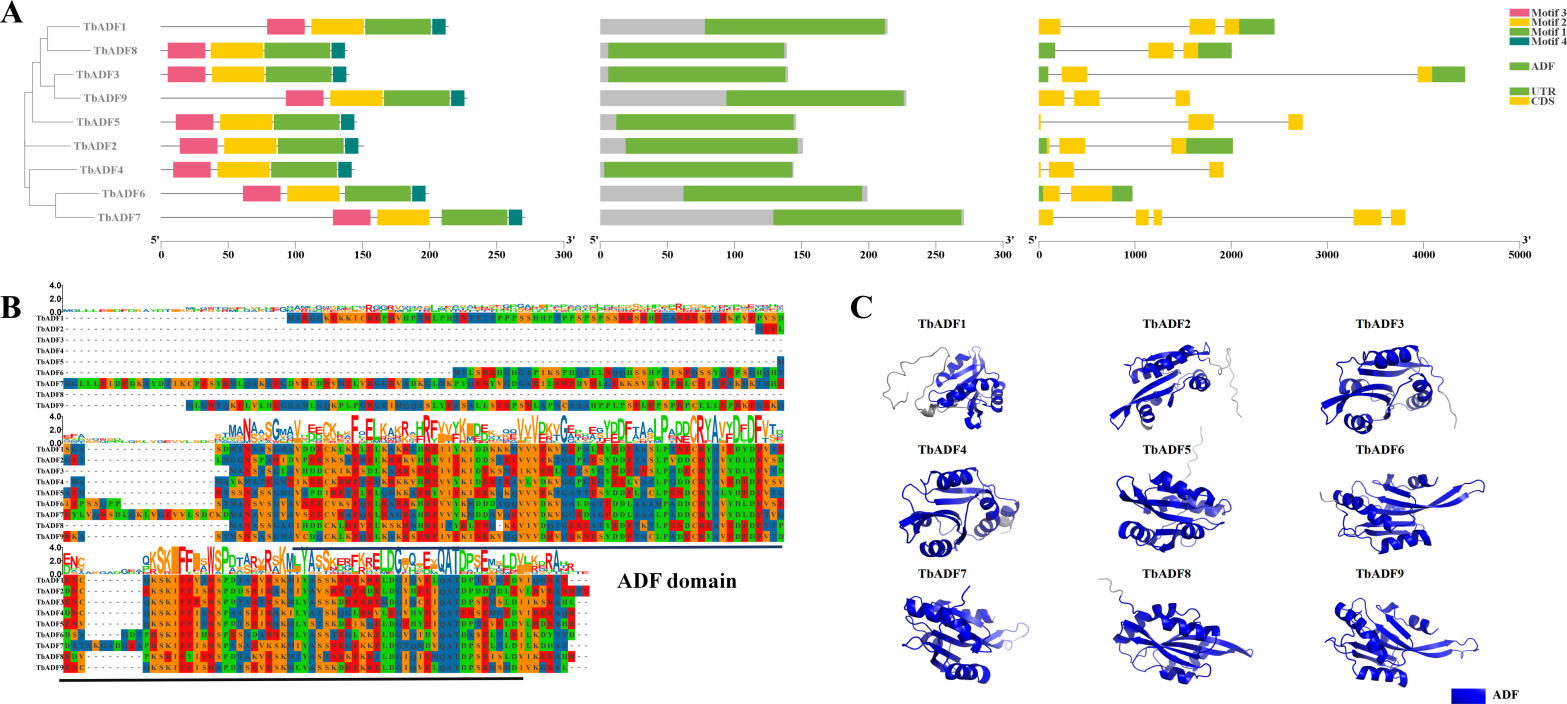
Conservation of ADF gene protein motifs, domains, and gene structure in *Triticum monococcum* L. subsp. *aegilopoides* **(A)**; Protein sequence alignment **(B)**; Protein tertiary structure **(C)**.

Gene structure analysis revealed a relatively conserved exon-intron architecture among the nine *TbADFs*. Three genes contained two exons, five had three exons, and *TbADF7* exhibited the most complex structure with five exons (**Figure 3A**). This conserved structural architecture suggests evolutionary stability within the ADF gene family. Motif analysis using the MEME Suite identified four conserved motifs shared by all *TbADFs*, predominantly located within the canonical ADF domain (**Figure 3A**). Multiple sequence alignment and tertiary structure modeling further confirmed the conserved positioning of the ADF domain across all family members (**Figure 3B**). Structural predictions generated by AlphaFold2 and visualized with PyMOL reinforced this conserved spatial configuration, highlighting the structural integrity of the ADF domain among *TbADFs* (**Figure 3C**).

**Figure 4:**
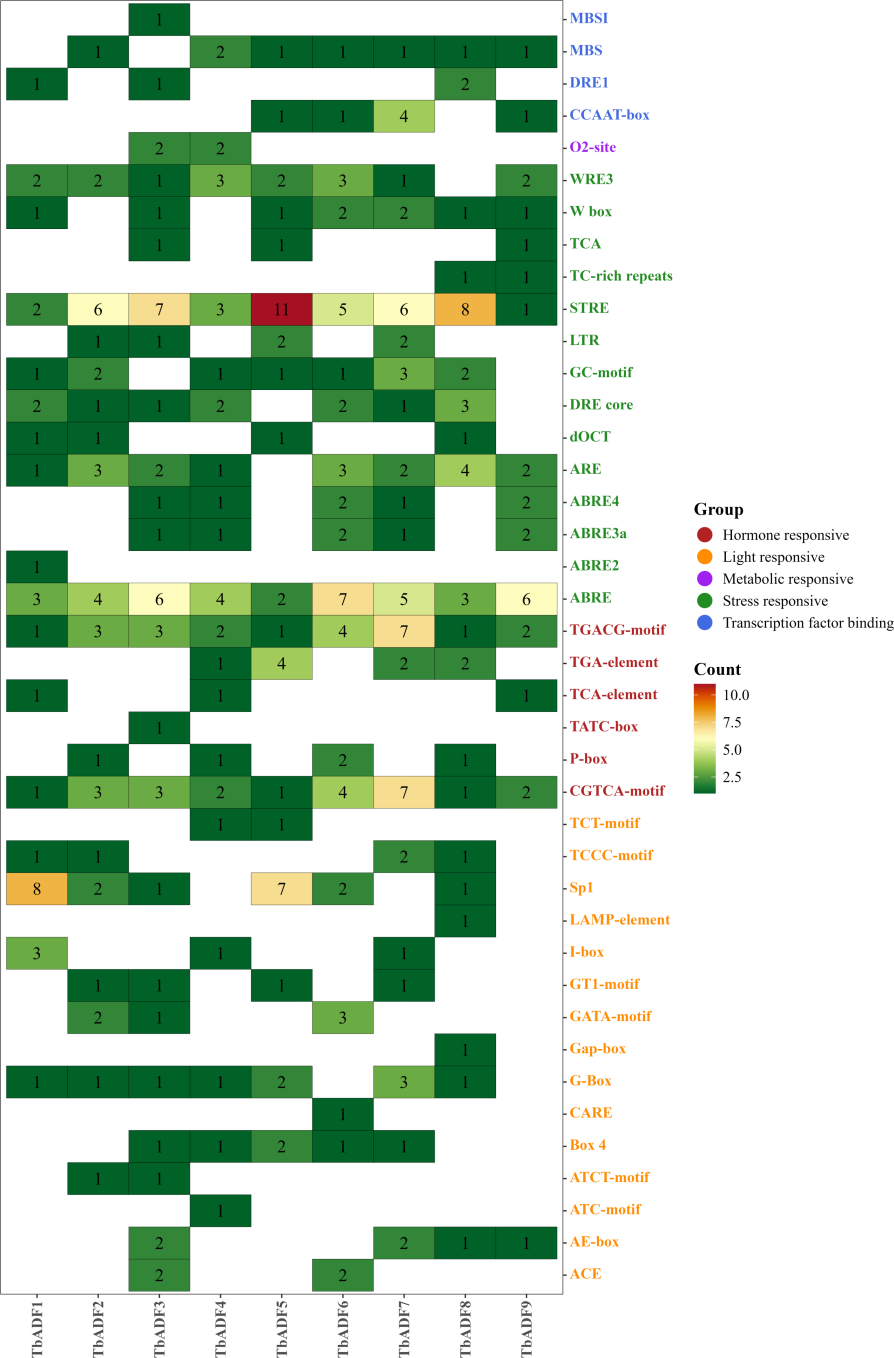
The number and functional classification of cis-acting elements in the *TbADFs*.

To explore regulatory potential, the 2,000 bp upstream promoter regions of *TbADFs* were analyzed using PlantCARE. Numerous cis-acting regulatory elements were detected, including those associated with growth, hormone signaling, and stress response (**Figure 4**; **Supplementary Table S3**). Notable elements included hormone-responsive motifs such as the abscisic acid response element (ABRE), jasmonic acid-responsive motifs (CGTCA-motif and TGACG-motif), auxin-responsive TGA-element, and gibberellin-responsive P-box. Additionally, stress-associated elements such as the low-temperature response element (LTR), drought-responsive DRE, and hypoxia-responsive motifs (GC-motif, ARE) were also identified. Collectively, these findings suggest that *TbADFs* are not only structurally conserved but are also likely involved in complex regulatory networks mediating hormonal and environmental stress responses. This highlights their potential roles in enhancing wheat adaptability to adverse environmental conditions.

### Prediction of catalytic active sites and binding pockets in *TbADFs*

**Figure 5:**
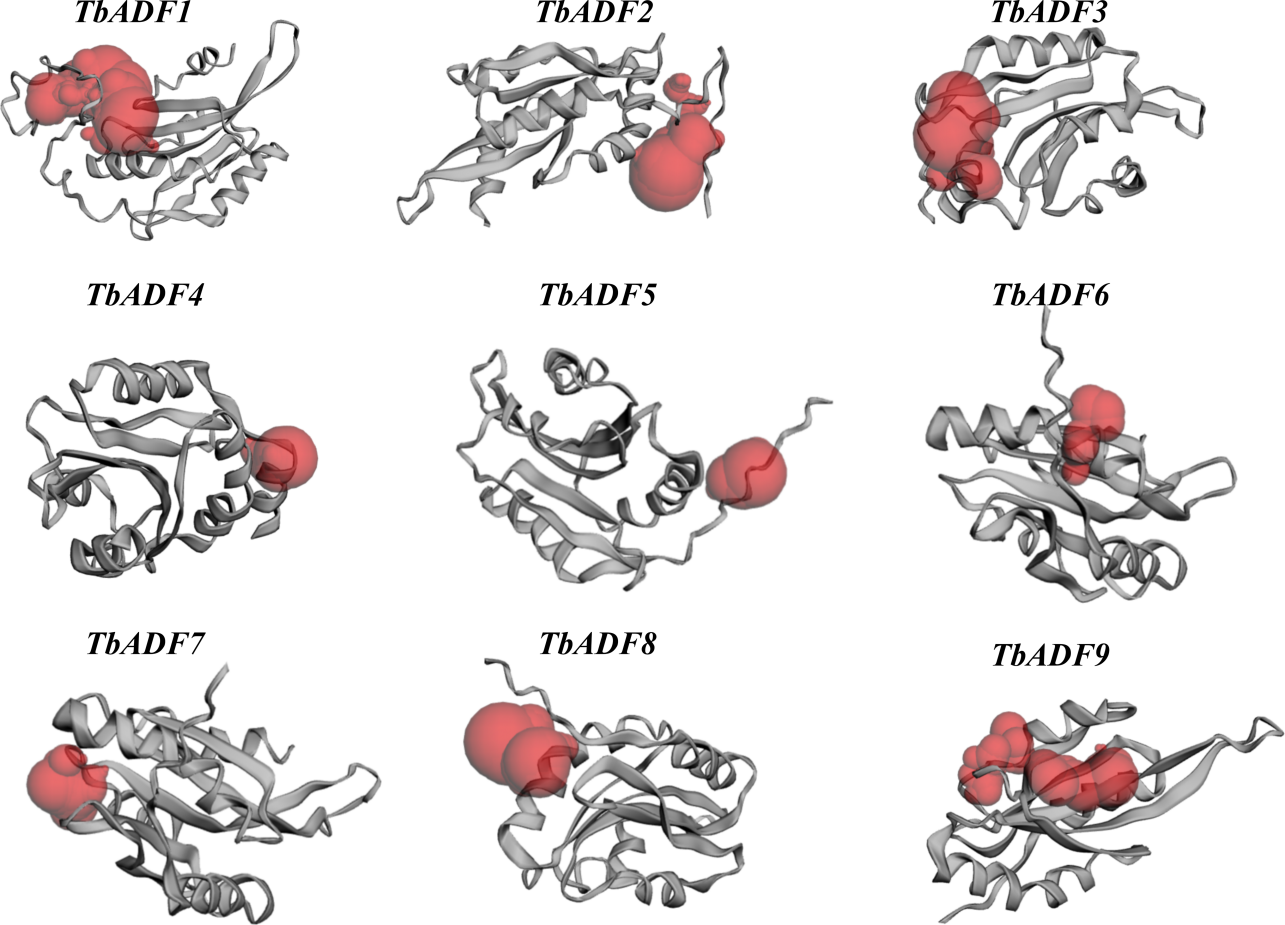
Predicted pocket binding sites of the *TbADFs*.

The detection of protein binding pockets is critical for understanding structural diversity and interaction specificity, particularly in predicting functional sites such as ligand-binding regions. To explore functional diversity among *TbADFs*, potential catalytic and ligand-binding sites were predicted using CASTp 3.0. Red-highlighted regions indicated the predicted binding pockets (**Figure 5**), and their surface areas and volumes were calculated (**Supplementary Table S4**). These binding sites are surrounded by key amino acid residues such as tyrosine (Tyr), phenylalanine (Phe), valine (Val), asparagine (Asn), lysine (Lys), alanine (Ala), aspartic acid (Asp), glutamic acid (Glu), isoleucine (Ile), and leucine (Leu), which are likely involved in mediating protein–ligand interactions and catalytic activity. The conservation of these residues supports their functional importance and potential roles in the stress-responsive behavior of ADFs.

### Prediction of protein-protein interactions in the *TbADFs*

**Figure 6:**
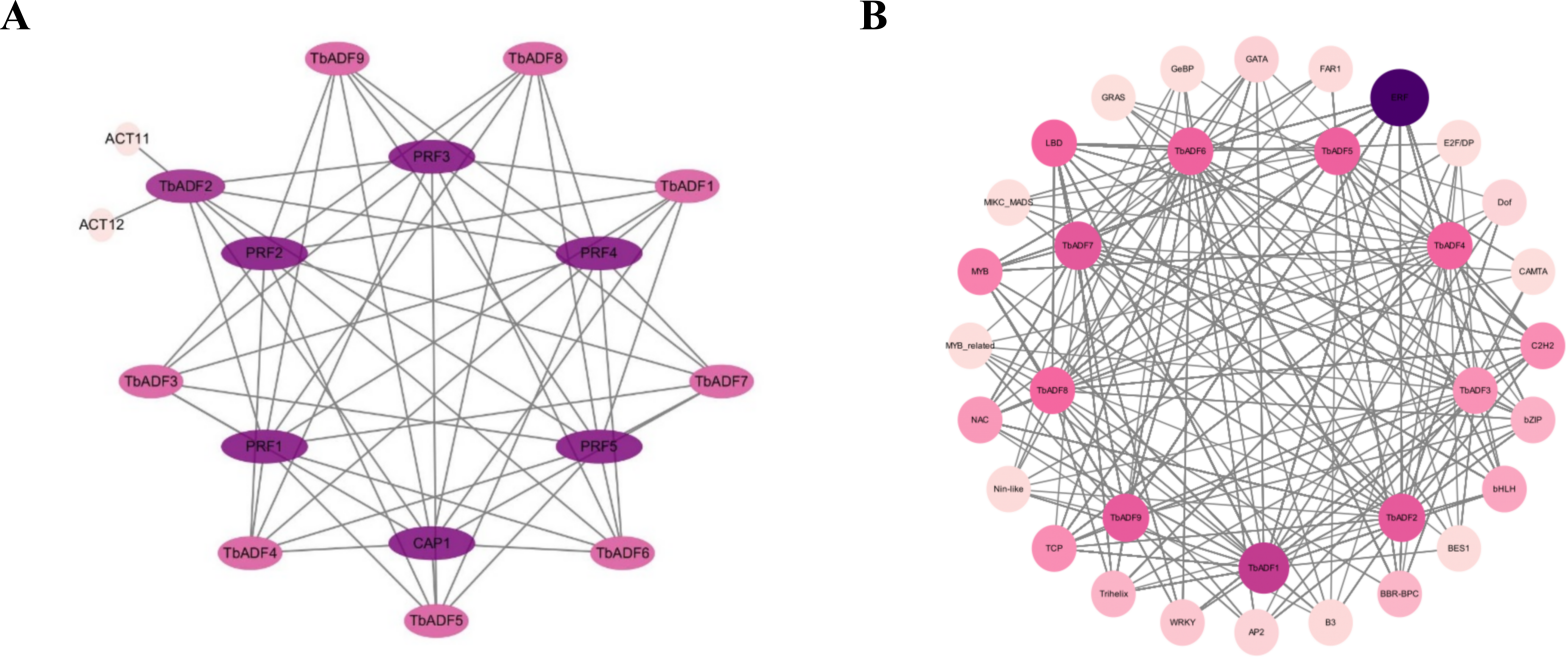
Predicted protein-protein interactions and transcription factor binding networks of *TbADFs* in *Triticum monococcum* L. subsp. *aegilopoides*. **(A)** Protein-protein interaction network of *TbADFs* with interacting partners. **(B)** Transcription factor binding network highlighting interactions between *TbADFs* and various transcription factor families.

To elucidate the protein interaction network of the ADF gene family in *Triticum monococcum* L. subsp. *aegilopoides*, nine TbADF protein sequences were analyzed using the STRING database with *A. thaliana* as the reference organism. (**Figure 6A**). These interacting proteins are implicated in critical cellular processes, including cytoskeletal dynamics, cell elongation, shape modulation, division, and migration, which collectively influence transpiration regulation, pathogen defense responses, and overall plant growth and development. The high connectivity of *TbADFs* with these partners, supported by a confidence score threshold of 0.7, suggests a conserved functional module that may underpin stress adaptation and developmental plasticity in wild einkorn wheat. This interaction network provides a foundation for future functional studies.

To further explore transcriptional regulation, promoter regions of the *TbADFs* genes were scanned for TF binding motifs using the FIMO (**Figure 6B**). This analysis revealed a complex regulatory network characterized by diverse TF interactions. Notably, members of the ERF family exhibited the highest binding site frequency, with a total of 2516 sites identified across multiple *TbADFs*, particularly in *TbADF1* and *TbADF7*. This suggests that ERF transcription factors may play a dominant role in modulating ADF gene expression, likely in response to abiotic stress signals, given the GC-rich nature of their target motifs. In addition, several other TF families, including LBD, MYB, TCP, and C2H2, also contribute to the regulatory architecture. Although these families are less abundant than ERFs, their presence suggests auxiliary roles in fine-tuning ADF gene expression under specific developmental or stress-related conditions.

### Codon usage bias analysis of *TbADFs*

**Figure 7:**
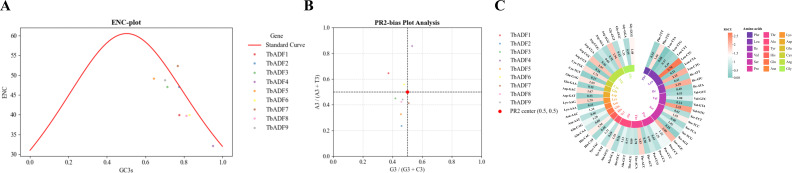
Codon usage bias analysis of *TbADFs* in *Triticum monococcum* L. subsp. *aegilopoides.* **(A)** ENC-plot showing the ENC versus GC3s, with the standard curve indicating expected ENC under neutral evolution; data points represent *TbADF1* to *TbADF9*, highlighting moderate codon usage bias. **(B)** PR2-bias plot illustrating the relationship between A3/(A3+T3) and G3/(G3+C3) for each *TbADFs*, with the central point (0.5, 0.5) indicating balanced nucleotide usage; data points cluster near this center, suggesting combined effects of mutation and selection. **(C)** RSCU heatmap displaying RSCU values for *TbADFs*, with color gradients indicating codon preferences (red for high, blue for low); amino acids are labeled with their corresponding codons, revealing a preference for G/C-ending codons.

The codon usage bias of the *ADF* gene family in *Triticum monococcum* L. subsp. *aegilopoides* was analyzed to explore the evolutionary pressures shaping gene sequence composition. The ENC plot (**Figure 7A**) showed that *ADF* genes exhibit ENC values ranging from 39.7 to 52.3. Most values fall below the expected standard curve under neutral evolution, indicating a moderate codon usage bias predominantly influenced by GC content at the GC3s, which ranged from 0.644 to 0.829 (**Supplementary Table S5**).

The PR2-bias plot (**Figure 7B**) demonstrated an approximately balanced distribution between A3 and T3, and between G3 and C3, with most data points clustering near the central point (0.5, 0.5). This pattern suggests that both mutational pressure and natural selection contribute to shaping codon usage in *TbADFs*.

A heatmap of RSCU values (**Figure 7C**) further highlighted a preference for G/C-ending codons across the *ADF* family, consistent with the GC-rich genomic context of *T. monococcum* subsp. *aegilopoides*. Notably, codons such as GGC (Glycine) and UGC (Cysteine) were overrepresented, implying potential optimization for translation efficiency. This bias may be linked to tRNA abundance or a selective advantage for efficient protein synthesis, particularly under stress conditions, aligning with the known role of ADF proteins in cytoskeletal dynamics and stress response.

### Analysis of DNA methylation levels in *TbADF*s

This analysis was based on high-quality, genome-wide methylation data for *Triticum monococcum* L. subsp. *aegilopoides* accession TA299, which we obtained from Ahmed et al. (2023). The final dataset comprised 221,159,329 CpG sites with a total sequencing depth of 19,524,541,735 and an average coverage of 88.28× per site. Using this high-coverage dataset, we calculated average methylation frequencies for all CpG sites within each defined gene region. The analysis of CpG methylation frequencies in the promoter and gene body regions of nine ADF genes (*TbADF1* to *TbADF9*) revealed significant variation in methylation levels (**Supplementary Table S6**), with promoter methylation ranging from 0.0907 (*TbADF2*) to 0.3053 (*TbADF9*) and gene body methylation from 0.0797 (*TbADF2*) to 0.2740 (*TbADF1*). The mean promoter methylation (0.1923) was slightly higher than the mean gene body methylation (0.1823), suggesting a potential regulatory role of promoter methylation in gene expression. Notably, *TbADF9* exhibited the highest methylation levels in both regions (0.3053 and 0.2644, respectively), which may indicate a repressive epigenetic state, while *TbADF2* displayed the lowest levels (0.0907 and 0.0797).

### Gene expression analysis of *TbADFs* in different tissues

**Figure 8:**
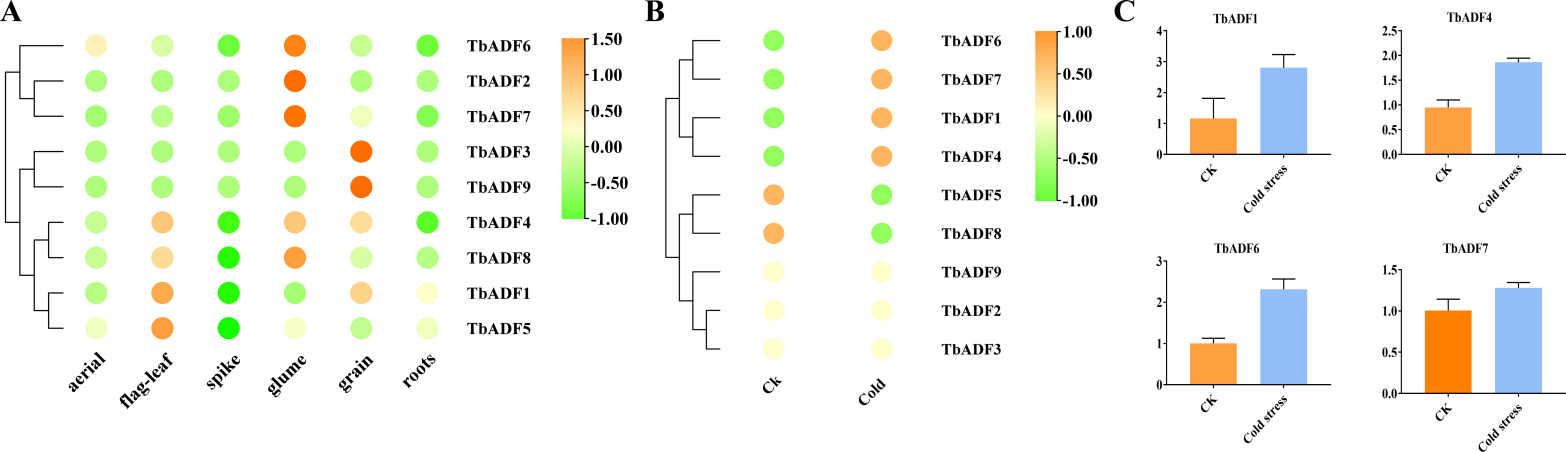
Expression profiles and cold stress response of *TbADF* genes in *Triticum monococcum* L. subsp. *aegilopoides*. **(A)** Expression profiles of *TbADF* genes across six tissues: aerial, flag leaf, spike, glume, grain, and roots. **(B)** Expression changes of *TbADF* genes under cold stress treatment based on transcriptome data. **(C)** qPCR validation of four selected *TbADF* genes under cold stress conditions.

Transcriptomic analysis across six tissues of *Triticum monococcum* L. subsp. *aegilopoides* revealed distinct tissue-specific expression patterns among the nine *TbADFs* (**Figure 8A**). *TbADF2* and *TbADF7* exhibited predominant expression in the glumes, whereas *TbADF3* and *TbADF9* were highly expressed in the grains, with low expression in other tissues. The remaining genes showed broader expression profiles: *TbADF1* was mainly expressed in both flag leaves and grains; *TbADF4* displayed elevated expression in flag leaves, glumes, and grains; and *TbADF5* and *TbADF8* were primarily expressed in flag leaves, glumes, and roots. These expression patterns indicate that *TbADFs* are functionally diversified, contributing to tissue-specific roles during the growth and development of wild einkorn wheat.

### Transcriptomic response of *TbADFs* to cold stress

Transcriptomic profiling following a 24-hour cold treatment at 4°C revealed distinct differential expression patterns among the *TbADFs*. Notably, *TbADF2*, *TbADF3*, and *TbADF9* exhibited no detectable expression under either control or cold-stress conditions, suggesting their non-involvement in the immediate cold response (**Figure 8B**). In contrast, the remaining six ADF genes were cold-responsive: *TbADF5* and *TbADF8* showed downregulation, whereas *TbADF1*, *TbADF4*, *TbADF6*, and *TbADF7* were significantly upregulated. Among them, *TbADF6* displayed the most dramatic induction, with TPM increasing from 29.07 to 300.01, highlighting its potential central role in cold stress adaptation. To validate these transcriptomic findings, expression analysis of *TbADF1*, *TbADF4*, *TbADF6*, and *TbADF7* was conducted, confirming consistent upregulation of all four genes under cold stress conditions (**Figure 8C**). This concordance between transcriptome data and expression validation strongly supports their involvement in the cold response of wild einkorn wheat.

**Figure 9:**
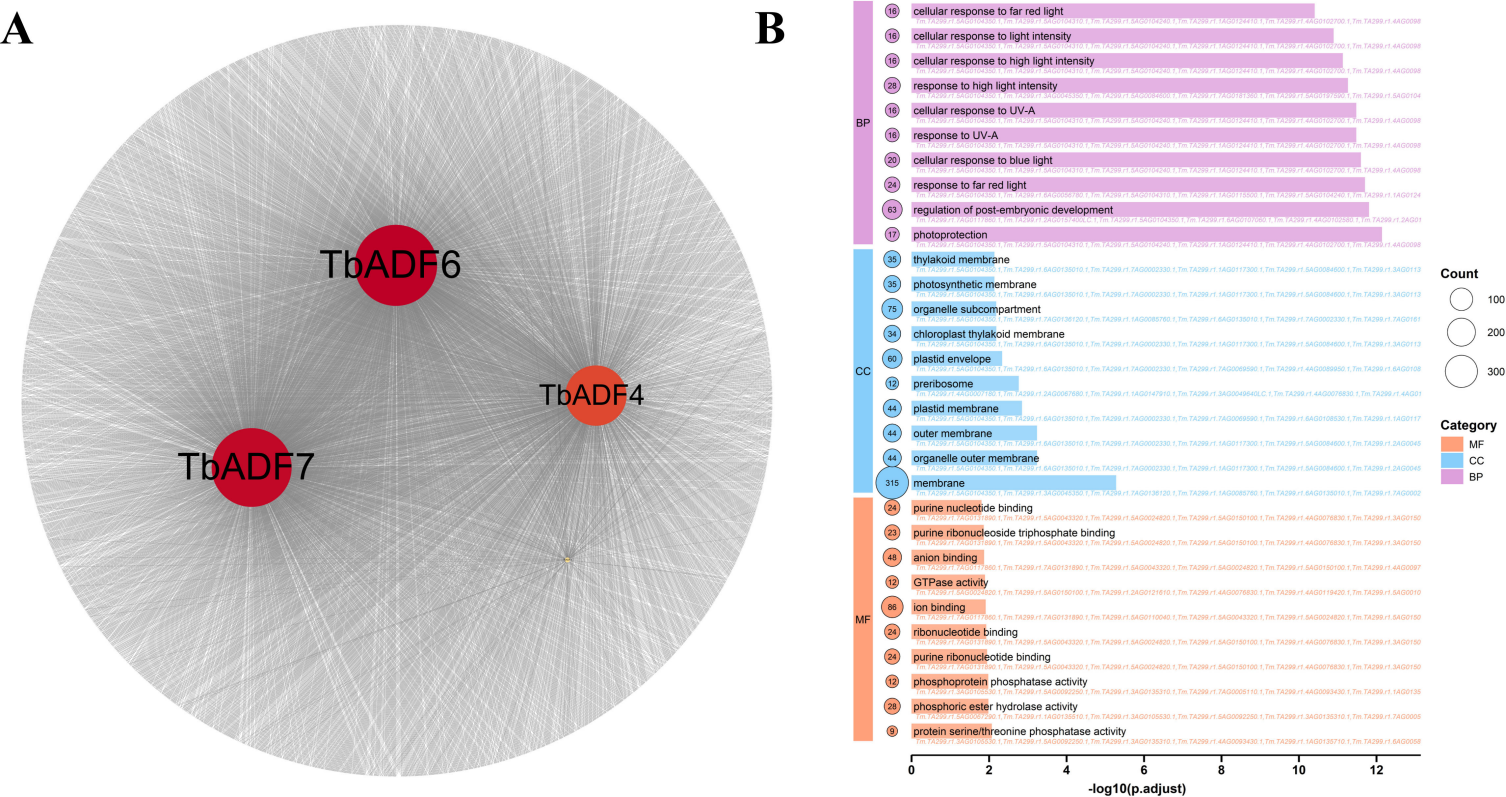
Co-expression network and GO enrichment analysis of genes highly correlated with four ADF genes under cold stress in *Triticum monococcum* L. subsp. *aegilopoides*. **(A)** Co-expression network illustrating interactions of genes highly correlated with *TbADF1*, *TbADF4*, *TbADF6*, and *TbADF7* (highlighted in red), with node size and connectivity reflecting the strength of associations. **(B)** Bar plot of GO enrichment analysis showing significantly enriched biological processes (BP), cellular components (CC), and molecular functions (MF) among co-expressed genes, with -log10(p-adjust) values indicating statistical significance and color coding by category.

To further dissect the molecular mechanisms underlying this response, we focused on the four upregulated genes, *TbADF1*, *TbADF4*, *TbADF6*, and *TbADF7*. Pearson correlation analysis (r > 0.9, *p*< 0.001) identified strongly co-expressed genes under cold stress: 103 for *TbADF1*, 1,269 for *TbADF4*, 1,706 for *TbADF6*, and 1,657 for *TbADF7* (**Figure 9A**). To elucidate the biological relevance of these co-expression networks, we performed GO enrichment analysis (**Figure 9B**).

In the MF category, terms such as purine ribonucleoside triphosphate binding (GO:0035639) and phosphoprotein phosphatase activity (GO:0004721) were significantly enriched, implicating roles in energy metabolism and phosphorylation-dependent signal transduction during cold stress. In the CC category, enrichments for membrane (GO:0016020) and organelle outer membrane (GO:0031968) suggest localization of co-expressed proteins to structural membrane sites, possibly mediating signal transmission and maintaining cellular integrity under stress. BP terms such as maturation of LSU-rRNA (GO:0000470) and intracellular chemical homeostasis (GO:0055082) point to enhanced ribosome biogenesis and metabolic homeostasis, key mechanisms for sustaining protein synthesis and stability during stress. Furthermore, enrichments in response to fatty acid (GO:0070542) and cellular response to endogenous stimulus (GO:0071495) underscore the involvement of lipid signaling and hormone-regulated pathways, both critical for cold adaptation through modulation of membrane fluidity and internal signaling cascades. Collectively, these findings highlight the intricate regulatory and functional roles of cold-inducible ADF genes, particularly *TbADF6*, in orchestrating a coordinated molecular response to low-temperature stress in wild einkorn wheat.

### Subcellular localization of *TbADF6* protein

**Figure 10:**
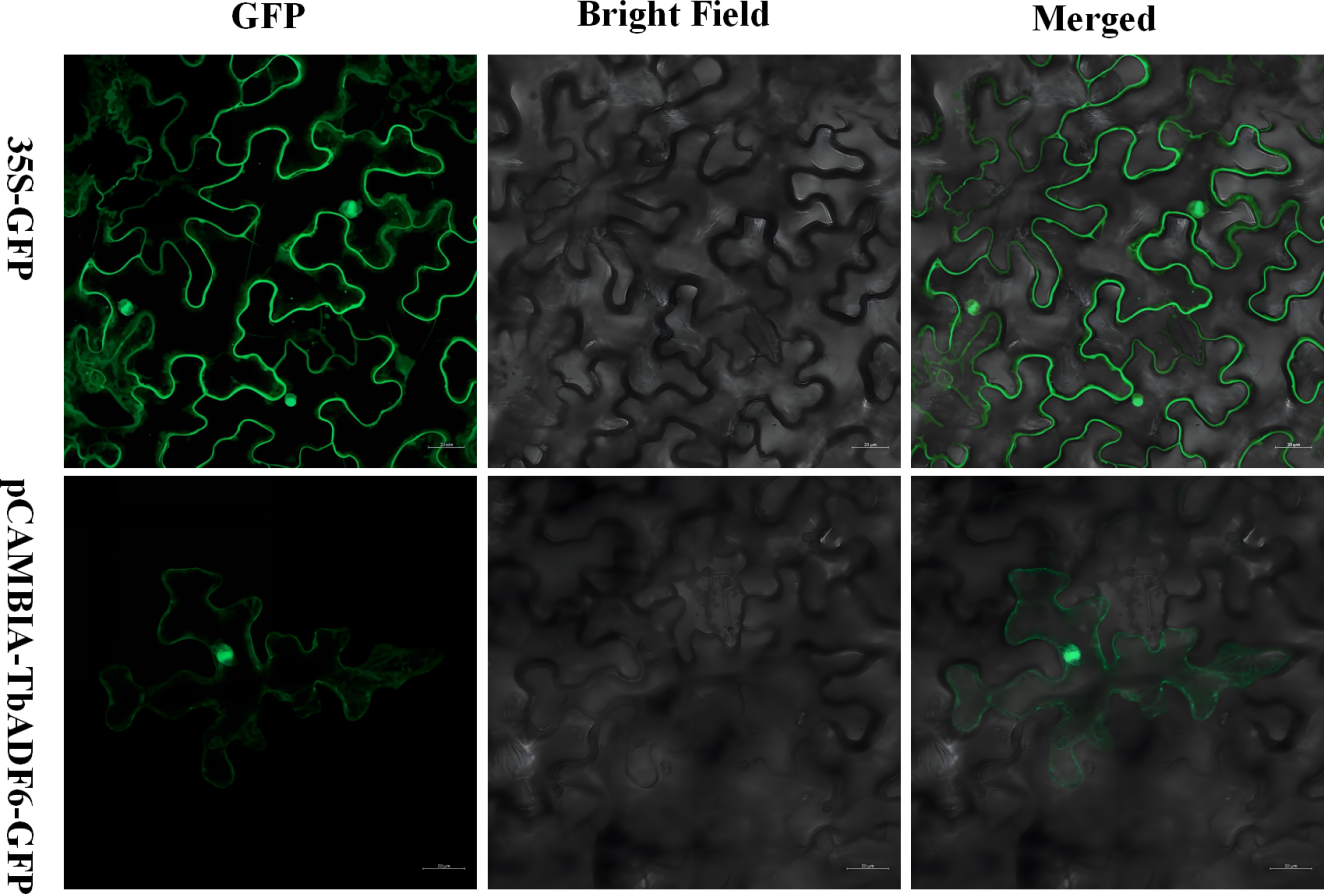
Subcellular localization analysis of *TbADF6* in *Nicotiana tabacum* leaves; 35S-GFP is the negative control. Scale bar: 20 µm.

To investigate the subcellular localization of *TbADF6*, a GFP-tagged fusion construct was generated and transiently expressed in tobacco leaves via Agrobacterium-mediated transformation. Confocal laser scanning microscopy showed that the GFP-ADF fusion protein was mainly localized in the nucleus and cytoplasm (**Figure 10**), which is consistent with the subcellular localization prediction (**Supplementary Table S2**). These results suggest that *TbADF6* protein may function in both compartments.

## Discussion

ADFs are key regulators of cytoskeletal dynamics in plants, influencing essential cellular processes such as morphogenesis, intracellular trafficking, and responses to abiotic stresses. Increasing evidence highlights their critical role in enhancing plant resilience under cold stress by modulating actin filament organization and signaling pathways (Staiger, 2000; Staiger and Blanchoin, 2006). In this study, we present a comprehensive characterization of the *ADF* gene family in *Triticum monococcum* L. subsp. *aegilopoides*, a wild diploid wheat species valued for its inherent stress tolerance. Through an integrative approach combining genomic, structural, and transcriptomic analyses, we identified nine *TbADFs* and explored their potential involvement in cold stress adaptation. Our findings provide new insights into the evolutionary conservation and functional diversification of *TbADFs* and lay the groundwork for future studies on cytoskeletal regulation under stress conditions.

The discovery of nine *TbADFs* in *T. monococcum* subsp. *aegilopoides* is consistent with the ADF gene family sizes reported in other monocots, such as rice and maize, which harbor 11 and 15 ADF genes, respectively (Yang et al., 2024). Phylogenetic analysis classified these *TbADFs* into four subgroups, a pattern conserved across plant species, suggesting an ancient diversification of ADF functions prior to monocot-dicot divergence (Ruzicka et al., 2007). The chromosomal distribution, with a notable cluster of *TbADF6*, *TbADF7*, and *TbADF8* on chromosome 5A^b^, hints at tandem duplication events, a common mechanism for gene family expansion and functional specialization in plants (Cannon et al., 2004). Such duplication events can provide raw genetic material for evolutionary innovation, allowing duplicated genes to diverge in expression patterns or molecular function (Mondal et al., 2016). The physical proximity of *TbADF6*, *TbADF7*, and *TbADF8* suggests they arose from local duplication, and preliminary expression data indicate that these genes exhibit differential tissue-specific expression, supporting the hypothesis of functional divergence. This likely reflects subfunctionalization or neofunctionalization processes that enable more refined regulation of actin dynamics in response to developmental or environmental cues (Panchy et al., 2016). Collinearity analysis with *T. urartu*, *T. turgidum*, and *T. aestivum* further revealed a high degree of conservation across wheat species, despite differing ploidy levels. This preservation through polyploidization events underscores the indispensable role of ADFs in cytoskeletal regulation and stress responses, likely maintained by purifying selection (Adams and Wendel, 2005).

Structural analyses revealed that the *TbADFs* possess conserved exon–intron structures and motif organizations, particularly within the actin-binding ADF domain, which is central to actin filament severing and depolymerization (Lappalainen et al., 1998). 3D structure prediction and binding site analysis via AlphaFold2 and CASTp identified conserved residues, such as tyrosine and lysine, within potential actin-interaction pockets, mirroring functional features described in *AtADFs* (Dong et al., 2001). Additionally, codon usage analysis revealed a strong preference for G/C-ending codons, which may enhance translation efficiency under stress conditions, an adaptive feature commonly observed in stress-responsive genes (Sharp and Li, 1987).

Transcriptomic analysis across six tissues of *Triticum monococcum* L. subsp. *aegilopoides* revealed distinct tissue-specific expression patterns among the nine *TbADFs*, underscoring their functional diversification in wild einkorn wheat. These genes encode proteins critical for regulating actin cytoskeleton dynamics, which are essential for key cellular processes such as division, expansion, and responses to abiotic stress. Notably, *TbADF2* and *TbADF7* exhibiting high expression in glumes, suggesting specialized roles in cytoskeletal remodeling during the development of these protective structures, which are vital for safeguarding reproductive tissues under fluctuating environmental conditions (Whitehead and Crawford, 2005). *TbADF3* and *TbADF9* were more abundant in grains, likely by facilitating actin-mediated nutrient transport and cell wall biosynthesis (Hussey et al., 2006). *TbADF1* was primarily expressed in flag leaves and grains, implying a role in coordinating cytoskeletal dynamics to support resource allocation during grain maturation (Hussey et al., 2002). *TbADF4* displayed elevated expression in flag leaves, glumes, and grains, suggesting a versatile function in metabolically active tissues. *TbADF5* and *TbADF8* were predominantly expressed in flag leaves, glumes, and roots, likely contributing to root growth and nutrient transport (Jiang et al., 1997). Under cold stress conditions, *TbADF1*, *TbADF4*, *TbADF6*, and *TbADF7* were significantly upregulated, with *TbADF6* showing the most prominent induction. Given the known function of ADFs in actin remodeling, these genes may help stabilize cytoskeletal structure, support vesicle trafficking, or preserve membrane organization during cold-induced cytoplasmic changes (Örvar et al., 2000).

Co-expression analysis of cold-induced *TbADFs* revealed networks enriched in genes associated with energy metabolism, signal transduction, and membrane integrity. Enriched GO terms such as purine ribonucleoside triphosphate binding and membrane organization underscore the cytoskeleton’s central role in energy-dependent stress signaling. PPI predictions using STRING uncovered potential interactions with profilins (PRFs) and cyclase-associated proteins (CAP1), key components of actin turnover and cytoskeletal reorganization (Pollard and Cooper, 2009). These interactions highlight the functional integration of *TbADFs* within broader stress adaptation networks.

The transcriptional response of the *TbADFs* to cold stress indicates that specific members are tightly regulated in response to low-temperature signals, suggesting functional divergence within this family. This variability aligns with previous findings in wheat, where ADFs exhibit differential expression under abiotic stresses (Xu et al., 2021). The complete transcriptional silence of *TbADF2*, *TbADF3*, and *TbADF9* under both control and cold-treated conditions may reflect either spatial or developmental specificity, pseudogenization, or functional redundancy that renders them unresponsive to acute cold stimuli. Such silencing could be indicative of evolutionary adaptations, as observed in other plant species where non-responsive gene family members serve niche roles (Moore and Purugganan, 2005). In contrast, the upregulation of *TbADF1*, *TbADF4*, *TbADF6*, and *TbADF7* underscores their potential involvement in early cold stress signaling or adaptation processes. Among these, *TbADF6* exhibited the most striking induction, suggesting it may act as a primary cold-responsive regulator within the ADF family in wild einkorn wheat.

Our integrative analysis suggests that multiple *TbADFs* collaboratively contribute to *Triticum monococcum* L. subsp. *aegilopoides*’s cold stress adaptation through both shared and distinct regulatory functions. GO enrichment of highly co-expressed partners for *TbADF1*, *TbADF4*, *TbADF6* and *TbADF7* indicates their involvement in diverse biological processes including ribonucleoprotein complex biogenesis, hormone metabolic pathways, photosynthetic membrane organization, and intercellular signaling. For example, *TbADF1* shows strong associations with nucleolar and ribonucleoprotein complex functions, suggesting roles in fundamental growth regulation, while *TbADF4* and *TbADF7* are enriched in pathways related to phosphatase activity, RNA helicase activity, and photoprotection. *TbADF6* is embedded in a dense regulatory network, sharing expression patterns with over 1,700 genes, implying its participation in broader stress-responsive pathways. This network density aligns with findings in rice, where hub genes under cold stress coordinate extensive gene interactions (Byun et al., 2021). GO enrichment of these co-expressed genes unveiled biological processes related to membrane structure, phosphorylation, ribosome maturation, and intracellular homeostasis—core components of cellular resilience to cold stress (Thomashow, 1999).

Similarly, recent studies in wheat identified 25 ADF genes, with *TaADF16* strongly upregulated under cold acclimation and freezing. Overexpression of *TaADF16* in Arabidopsis improved freezing tolerance by reducing ion leakage and increasing survival through better ROS scavenging and membrane protection. It also activated cold-responsive genes like *CBF1*, *COR15A*, and *RD22*, indicating involvement in the conserved ICE-CBF-COR pathway. Other *TaADFs*, such as *TaADF13*, *TaADF17*, and *TaADF22*, also respond to cold stress, suggesting a coordinated ADF gene network in cold adaptation (Xu et al., 2021). Notably, *TbADF6* is located on chromosome 5A^b^ in *Triticum monococcum* L. subsp. *aegilopoides* and shows conserved synteny with *TaADF16* on 5A, *TaADF18* on 5B, and *TaADF22* on 5D in hexaploid wheat. This collinearity indicates that *TbADF6* is the orthologous counterpart of these cold-responsive *TaADFs*, suggesting functional conservation and evolutionary retention of this locus across wheat genomes. The presence of homologous genes on chromosomes 5B and 5D reflects the two rounds of polyploidization events in wheat evolution (Ramírez-González et al., 2018), which generated additional homeologous copies of *TbADF6* in the B and D subgenomes. The syntenic relationship underscores the importance of *TbADF6* as a likely key regulator in cold adaptation, maintained across multiple wheat subgenomes through evolutionary history. Additionally, terms related to fatty acid response and endogenous stimulus perception suggest that lipid-mediated signaling and hormonal regulation may work in concert with *TbADFs*-mediated actin remodeling to maintain membrane integrity and initiate adaptive signaling under cold conditions. These processes are known to be critical in plants for maintaining cellular homeostasis under low temperatures. Together, these findings indicate that cold-inducible *TbADFs*, particularly *TbADF6*, play central roles in orchestrating cytoskeletal and signaling reprogramming during early cold response. This highlights ADF genes as potential targets for genetic manipulation aimed at improving cold tolerance in wheat and other cereals.

## Data Availability

The datasets presented in this study can be found in online repositories. The names of the repository/repositories and accession number(s) can be found in the article/**Supplementary Material**.
